# Effects of early intervention of swallowing therapy on recovery from dysphagia following stroke

**Published:** 2015-07-06

**Authors:** Jalal Bakhtiyari, Payam Sarraf, Noureddin Nakhostin-Ansari, Abbas Tafakhori, Jeri Logemann, Soghrat Faghihzadeh, Mohammad Hossein Harirchian

**Affiliations:** 1 Department of Speech Therapy, School of Rehabilitation, Tehran University of Medical Sciences, Tehran, Iran; 2 Iranian Center of Neurological Researches AND Department of Neurology, School of Medicine, Tehran University of Medical Sciences. Tehran, Iran; 3 Department of Physiotherapy, School of Rehabilitation, Tehran University of Medical Sciences, Tehran, Iran; 4 Departments of Neurology and Otolaryngology-Head and Neck Surgery Feinberg, School of Medicine, Northwestern University, Evanston, IL; 5 Department of Biological Statistics and Epidemiology, School of Medicine, Zanjan University Medical Sciences, Zanjan, Iran

**Keywords:** Stroke, Dysphagia, Speech Therapy

## Abstract

**Background: **Dysphagia is common after stroke. The onset time of swallowing rehabilitation following stroke has an important role in the recovery of dysphagia and preventing of its complications, but it was either highly variable or was not stated in previous trials. The aim of this study was investigation effects of onset time of swallowing therapy on recovery from dysphagia following stroke.

**Methods:** Sixty dysphagia patients due to stroke range of age 60-74 (67.1 ± 3.8), participated in this randomized clinical trial study. The patients allocated in Early, Medium and Late groups, on the base of initiation of swallowing therapy after the stroke. After basic clinical and video fluoroscopic swallowing study assessments, traditional swallowing therapy was initiated 3 times per week for 3 months. The outcome measures were North-Western dysphagia patient check sheet, functional oral intake scale, video fluoroscopy, and frequency of pneumonia. Statistical analysis was done by repeated measure ANOVA, Bonferroni and χ^2^ tests.

**Results: **Three groups of patients in terms of demographic and clinical characteristics were similar in the pre-treatment P > 0.050. Onset time of swallowing therapy after stroke was effective on swallowing recovery on the main outcome variables. So that in first group patients, recovery was rather than other groups P < 0.050. Furthermore, the frequency of pneumonia in the early group was less than other groups and in the early group no patients experienced pneumonia P = 0.002.

**Conclusion: **Our data suggested that early interventions for dysphagia in stroke have an important role in recovery from dysphagia and prevention of complications like aspiration pneumonia.

## Introduction

Swallowing, as the first phase of digestion, is one of the most complicated neuromuscular processes of the central nervous system. It involves multiple areas of the brain and a series of voluntary and involuntary muscular contractions.

Oropharyngeal dysphagia is a highly prevalent clinical condition among stroke patients, but the prevalence of dysphagia is different in various studies, because of differences in the definition of dysphagia, the method of assessing swallowing function, the timing of swallowing assessment after stroke, and the number and type of stroke patients studied.^1^^-^^3^ Overall, swallowing disorders (dysphagia with or without aspiration) are seen in about half (55%) of all stroke patients admitted to hospital.^4^^-^^7^

The presence of dysphagia can itself cause medical, psychosocial, and economic complications in stroke patients. A medical complication of dysphagia includes aspiration pneumonia, malnutrition, significant weight loss, and dehydration.^8^^-^^12^ Another complication of dysphagia in stroke patients is psychosocial because eating is a pleasurable and social activity, and inability to eat normally may affect patient morale and quality-of-life.^13^^,^^14^

Complications due to dysphagia especially include pneumonia, and managing infection also increases healthcare costs by increasing the length of hospital stay and increasing the need for expensive respiratory and nutritional support.^7^

To prevent and minimize these complications, diagnosis and management of dysphagia must be done as soon as possible by a trained speech-language pathologist.^15^

The current treatment of dysphagia in patients with stroke is the traditional swallowing therapy by a speech therapist. Compensatory approaches and rehabilitative methods are included in this therapy.

Compensatory approaches include: enteral feeding by means of a nasogastric tube or by percutaneous endoscopic gastrostomy, modification of food consistency, postural correction to facilitate bolus transition, reducing rate of eating and ensuring oral hygiene by conventional oral care.^16^ Other approaches are rehabilitative methods, including oral motor exercises; airway-protecting maneuver, thermal-tactile stimulation, and Shaker exercise.^16^^-^^18^ Recently, neuromuscular electrical stimulation, biofeedback, and transcranial magnetic stimulation have been used as techniques for swallowing therapy these techniques are modern swallowing therapy.^19^^-^^22^

The onset time of swallowing rehabilitation following stroke was either highly variable or was not stated in the most of investigations. In some studies, interventions have been initiated within 7 days after stroke^23^ or 24 h after stroke^24^ and between 4 and 6 weeks or even 3-6 months post-stroke.^25^^,^^26^ In the other hand, some studies have only focused on early intervention, and do not consider the time at which swallowing rehabilitation should be initiated for optimal recovery.^23^^,^^24^

The onset time of intervention following stroke is uncertain, and no completed randomized clinical trial (RCT), assessing this question was found. Thus, the aim of this study was to investigate the effect of onset time of traditional swallowing therapy, given by a speech therapist on recovery from dysphagia in stroke patients.

## Materials and Methods

Sixty patients, dysphasic due to stroke, in the age range of 60-75 (58.4 ± 7.8), participated in this RCT study. This study was single blind, because all patients included in this study were unaware of their allocation into treatment groups, but the speech pathologist who evaluated and treated the patients was aware of the group and the radiologist who performed video fluoroscopy was unaware of the allocated group.

**Figure 1:**
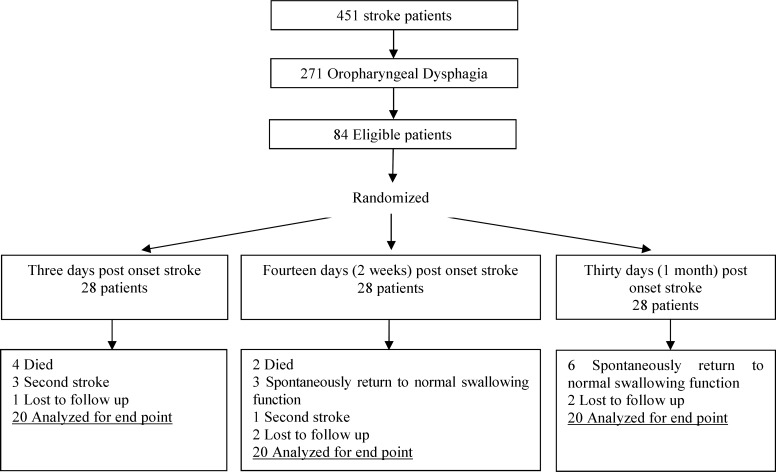
Clinical trial allocation information

Totally, 451 acute stroke patients presenting to the emergency neurology ward of our university hospital (Imam Khomeini Hospital) over a 2-year period (February 2012-January 2014) were screened for inclusion criteria of the study. Inclusion criteria in this study were as follows: clinical diagnosis of stroke which was confirmed by a neurologist presence of dysphagia which was assessed clinically by a speech-language pathologist and video fluoroscopically by a radiologist using standardized methods and diagnostic criteria; no history of swallowing treatment, pneumonia or head and neck surgery and other neurological or general disorders that can influence swallowing function. Of 451 stroke patients, 271 patients had dysphagia, and 84 patients were eligible for our study. 24 patients were excluded due to follow-up problems, and finally 60 patients were analyzed (Figure 1).

Randomization was undertaken using a block randomization technique. The patients were allocated into one of three groups according to the time of initiation of swallowing therapy after stroke including: (1) early initiation group (3 days after stroke); (2) medium group (2 weeks after stroke); and (3) late group (1-month after stroke).

The study protocol was approved by the ethics committee of Tehran University of Medical Sciences. Informed consent was obtained from each participant or the next of kin before any examination or intervention was conducted.

This study has been registered in www.irct.ir, number IRCT2013072514161N1.

All patients were screened by the North-western Dysphagia Patient Check Sheet, and dysphagia was diagnosed by a speech pathologist. In all patients after primary diagnosis of dysphagia, swallowing functions were assessed by functional oral intake scale (FOIS), and video fluoroscopy was done by an attending radiologist. For medium and late groups, screening of swallowing function was performed by a speech pathologist weekly before initiation of swallowing therapy. In this period of time, all patients were provided with the usual oral care and advices for feeding such as: precautions for safe swallowing, including positioning and slowed rate of feeding) by the speech pathologist. The swallowing difficulties of some of the patients in these groups resolved spontaneously, and they were excluded from the study. The diagnosis of pneumonia was based on: fever, a productive cough with purulent sputum and abnormal finding in chest examination and chest X-ray. For all patients, traditional swallowing therapy includes compensatory methods, direct swallowing therapy and swallowing maneuvers were given by the speech pathologist 3 times per week for 3 months. The type of swallowing therapy technique was based on the findings of the clinical examination and video fluoroscopy for all patients. After 2 months, clinical and video fluoroscopy examinations were done for all patients.

Outcome measures in this study included: (1) scores of the North-Western dysphagia patient check sheet; (2) Difficulties in oral or pharyngeal stages swallowing and presence of pharyngeal delay or aspiration according to the North-Western dysphagia patients check sheet (NWDPCS) and video fluoroscopy; (3) FOIS; (4) frequency of pneumonia; and (5) the number of sessions of swallowing therapy needed for improvement.

Statistical analysis of data was done by SPSS software for Windows (version 19.0, SPSS Inc., Chicago, IL, USA), by the use of parametric statistical tests (e.g., repeated measure ANOVA for comparison of normal data between three groups pre and post treatments data) and by non-parametric tests such as χ^2^ and Cochran tests.

## Results

Three groups of patients in terms of demographic (age, gender, site of lesions, and type of stroke) and clinical characteristics were similar in the pre-treatment P > 0.050 (Table 1).

**Table 1:** Comparison of demographic characteristic of participants

**Characteristics**		**Three days post ** **onset (n = 20)**	**Two weeks post ** **onset (n = 20)**	**One month post ** **onset (n = 20)**	**P**
Age (mean ± SD)		66.40 ± 4.09	67.15 ± 3.67	67.85 ± 3.97	0.508
Sex (%)	Male	13 (65)	14 (70)	16 (80)	0.247
	Female	7 (35)	6 (30)	4 (20)	
Site of lesion (%)	Right hemisphere	11 (55)	11 (55)	13 (65)	0.957
	Left hemisphere	7 (35)	7 (35)	5 (25)	
	Brain stem	2 (10)	2 (10)	2 (10)	
Type of stroke (%)	Ischemic	14 (70)	16 (80)	18 (90)	0.287
	Hemorrhagic	6 (30)	4 (20)	2 (10)	

SD: Standard deviation

**Table 2:** Effects of onset time of swallowing therapy on swallowing recovery (North Western dysphagia patients check sheet)

**Sources**	**Sum of squares**	**df**	**Mean squares**	**F**	**P**
Groups (onset time of swallowing therapy)	33.1	2	16.50	8.4	0.0001
Factor (pre/post treatment)	2764.8	1	2764.80	1529.2	< 0.0001
Groups × factor	8.1	2	4.07	2.2	0.1140

df: Degree of freedom

**Table 3:** Paired comparisons of swallowing recovery (North-Western dysphagia patients check sheet)

**Groups**	**Paired comparisons**	**Mean difference**	**P**
3 days after stroke	2 weeks after stroke	-1.150	0.002
	1-month after stroke	-1.070	0.003
2 weeks after stroke	3 days after stroke	1.150	0.002
	1-month after stroke	0.075	1.000
1-month after stroke	3 days after stroke	1.075	0.003
	2 weeks after stroke	-0.075	1.000

**Table 4:** Comparison of swallowing function among three groups after therapy

**Variables**	**Groups of patients**			
	**Three days post onset ** **(n = 20) (%)**	**Two weeks post ** **onset (n = 20) (%)**	**One month post ** **onset (n = 20) (%)**	**P**
Presence of an oral stage swallowing problem				
NWDPCS	0 (0)	0 (0)	3 (15)	0.043
VFSS	0 (0)	0 (0)	3 (15)	0.025
Presence of an, Pharyngeal Stage swallowing problem				
NWDPCS	1 (5)	3 (15)	5 (25)	0.028
VFSS	1 (5)	4 (20)	9 (45)	0.010
Presence of pharyngeal delay	1 (5)	4 (20)	5 (25)	0.045
Presence of aspiration				
NWDPCS	1 (5)	4 (20)	6 (30)	0.043
VFSS	2 (10)	5 (25)	12 (60)	0.002

NWDPCS: North western dysphagia patients check sheet; VFSS: Video fluoroscopic swallowing study

The data analyzed by repeated measure ANOVA and for paired comparisons between groups, we used of post hoc Bonferroni test. The data indicated that onset time of swallowing therapy after stroke was effective on swallowing recovery on the main outcome variable( mean scores of NWDPCS) P = 0.001 (Table 2), so that in first group patients, recovery was rather than other groups P < 0.050, but between medium and late groups swallowing recovery was not differences P > 0.050 (Table 3).Comparison of the frequency of types of swallowing disorders between three groups indicated differences between three groups P < 0.050 (Table 4). Furthermore, the frequency of pneumonia in the early intervention group was less than other groups and in the early group, no patients experienced pneumonia P = 0.002 (Table 5).

The number of swallowing therapy sessions in the early intervention group was (10.25 ± 1.91), in medium group was (17.40 ± 2.60) and in 1-month post stroke group was (32.3 ± 3.2) thus the number of swallowing therapy sessions in the early intervention group significantly lower than in the other two groups and overall the mean of swallowing therapy sessions was significantly different in the three groups P < 0.001. 

## Discussion

The results of our study indicate that the time of initiation of swallowing therapy after stroke has an important role in the recovery of swallowing function, the presence of aspiration pneumonia and the number of swallowing therapy sessions. As in early intervention group recovery of swallowing function was better than other group, but between medium and late groups was not different on the recovery of swallowing function. These findings are in agreement with those of Takahata et al., which showed that early intervention can improve oral feeding in patients with intracerebral hemorrhage; but the interventions of their study were oral care, changing position, and dietary modifications, compared to ours which included traditional swallowing therapy.^25^ The findings of our study are also consistent with those of Carnaby et al., which found that their intervention for dysphagia within the 1^st^ week after stroke improved swallowing function, but they considered the intensity of treatment rather than the time of initiation of it.^24^ Some studies in the field of swallowing therapy have investigated a method or approach for dysphagia in stroke patients; these therapeutic methods are initiated in acute, sub-acute or chronic periods post-stroke, and the results indicate the positive effects of swallowing therapy without considering the time of initiation of swallowing therapy.^25^^-^^27^

**Table 5:** Comparison of presence of pneumonia and number in three groups

**Pneumonia**	**Three days post ** **onset (n = 20) (%)**	**Two weeks post ** **onset (n = 20) (%)**	**One month post ** **onset (n = 20) (%)**	**P**
Presence of pneumonia (before treatment)	0 (0)	6 (30)	10 (50)	0.002
Presence of pneumonia (after treatment)	0 (0)	1 (5)	3 (15)	0.158

Another issue related to swallowing problems in stroke patients is the spontaneous recovery from dysphagia. Studies have suggested that the recovery from dysphagia spontaneously occurs soon after the stroke, taking between 2 and 4 weeks, and so some clinicians initiate intervention for dysphagia for 2 weeks or more after stroke.^28^ However, these studies have relied on bedside clinical examination to diagnose dysphagia and have only assessed swallowing function for short periods, such as 2 weeks after stroke.^29^ Meanwhile, long-term follow-up of swallowing disability at 6 months post-stroke clinically and video fluoroscopically showed clinical evidence of a swallowing abnormality in 50% of stroke survivors.^2^ Furthermore, nearly half of aspirations in patients with stroke are silent,^1^^,^^30^ and these have been associated with increased morbidity and mortality in many studies.^31^ Thus, intervention for dysphagia management at the proper time can reduce these pulmonary complications. The results of this study showed this positive effect because early detection and management of dysphagia by swallowing techniques can reduce aspiration in stroke patients. The results of this study are consistent with the principles of brain neural plasticity, such as “use it or lose it,” and “use it and improve it.”^32^

This study is the first RCT that considers onset time of swallowing disorders in stroke patients, but there are some limitations in this study, mainly the awareness of the speech therapist about the group of the allocated patients.

Another limitation of this study was that some patients were not followed up due to repeated stroke within the stage of assessment or treatment period.

## Conclusion

The results of this study indicated that early dysphagia detection, using validated screening and assessment tools by a speech therapist and a standard dysphagia program of early swallowing intervention, not only improves swallowing function in stroke patients but also reduces pulmonary complications. The time taken to return to a normal diet was also significantly shorter for patients assigned to early intervention on the base of number of swallowing function.

## Notes:

**How to cite this article: **Bakhtiyari J, Sarraf P, Nakhostin-Ansari N, Tafakhori A, Logemann J, Faghihzadeh S, et al. Effects of early intervention of swallowing therapy on recovery from dysphagia following stroke. Iran J Neurol 2015; 14(3): 119-24.
